# Oxford Medical Degrees

**Published:** 1834-04-01

**Authors:** 


### OXFORD MEDICAL DEGREES.

The following regulations regarding degrees in medicine have been lately issued by the University of Oxford, and are to come into operation after Trinity Term, 1834.

### DE TEMPORE ET EXERCITIIS REQUISITIS AD GRADUS IN MEDICINA CEPASSENDOS.

Quoniam exercitia pro Gradibus in Facultate Medicinæ præstanda hisce temporibus minime conveniunt, placuit Academiæ abrogare Statuta (scil. Tit. VI. Sect. v. Gratiarum formulas Tit. IX. Sect. iii. §. 4. Juramenti formam Tit. IX. Sect. vi. §. 2 et Tit. IX. Sect. ix. §. 4. §. 5. §. 6.) et in eorum locum nova hæc Statuta subrogare.

§ 1. *Quot anni, in studio Medicinæ ponendi, requirantur ad Gradum Baccolaurei in Medicina.* Statutum est, quod unusquisque antequam Baccalaureatum in Medicina consequatur, examen publicum subeat inter eos Candidatos qui primum gradum in Artibus vel in Jure Civili petunt, et, post examen illud, Medicinæ studio per triennium integrum (seil. 12 Terminos) operam dedisse teneatur.

Magistris vero in Artibus, vel Baccalaureis in Jure Civili, licebit, (si modo examen subierint infra §. 3 sancitum,) Baccalaureatum in Medicina capessere; cauto tamen nequis ad hunc gradum admittatur ante completos 28 Terminos a die matriculationis suæ computandos.

§ 2. *De Examinatione pro Gradu Baccalaurei in Medicina, et de Examinatoribus designandis, et juramento onerandis.* Statutum est, quod qui ad Baccalaureatum in Medicina promoveri cupit, priusquam ad supplicandum pro Gratia admittatur, examen subeat.

Huic igitur rei quo melius prospiciatur, Academiæ placuit ut tres in Medicinæ Facultate publici Examinatores constituantur; scilicet, Medicinæ Professor Regius (cujus semper erit huic negotio præesse,) duo itidem ex Acad. Oxon. in Medicina Doctores, a Vice-Cancellario singulis Examinationibus nominandi, et deinceps a Domo Convocationis approbandi. Quod si Professor Regius propter, gravem aliquam causam absentiæ veniam a Vice-Cancellario impetraverit; alium quendam in Medicina Doctorem Vice-Cancellarius in ejus locum substituet.

Singuli autem Examinatores antequam munera sua adeant, coram Vice-Cancellario, exigente Seniore Procuratore, juramentum præstabunt in hæc verba; viz.

Domine Doctor, tu jurabis, quod munus et officium Examinatoris publici in Facultate Medicinæ sedulo et fideliter pro virili exequeris, forma et modo per Statuta requisitis. *Resp.* Juro.

Item tu jurabis, quod, sepositis omni odio et amicitia, timore et spe, neque indigno cuivis testimonium perhibebis, neque digno denegabis. *Resp.* Juro.

Item tu jurabis, quod nullo modo revelabis suffragium quod vel ipse tuleris, vel alius quivis Examinator tulerit, de testimonio alicui perhibendo vel denegando. *Resp.* Juro.

§ 3. *De tempore, loco et modo Examinationis.*—

Semel quolibet anno, videlicet Hebdomada secunda pleni Termini Trinitatis, Examinatio in Facultate Medicinæ habeatur.

Huic Examinationi liceat Examinatoribus cum consensu Vice-Cancellarii locum aliquem idoneum assignare, modo ante Examinationem habendam triduo saltem de hac re Academiam certiorem faciant.

Instituatur examen in Theoria et Praxi Medicinæ, in Anatomice, Physiologia, Pathologia, et Materia Medica, insuper in Chemia et Botanice, quatenus eæ disciplinæ Artem Medicam illustrare videantur. Examinatoribus liberum esto, quemlibet Candidatum vel in hisce universis, vel in aliqua parte harum (prout ipsis satius visum fuerit) examinare. Porro nunquam non adjiciantur veteres illi Scriptores, Hippocrates, Aretæus, Galenus et Celsus, quorum duo ad minimum in omni examine semper adhibentor.

Cujuslibet Candidati examen, partim viva voce, partim scriptis, peragatur, et quantum fieri poterit, uno decursu vel saltem sine diei intervallo, absolvatur. Omnes Examinatores uniuscujusque examini per integrum tempus intersint, nisi aliquid in scriptis præstandum sit, quo in casu unus tantummodo adesse teneatur.

Inter examinandum, aut Latino aut Anglicano Idiomate uti licebit, prout Examinatoribus magis expedire videbitur.

Peracta demum Examinatione, Examinatores de Candidatis singulis inter se judicium ferent, et quemcunque dignum invenerint, et testimonium perhibebunt sub hac forma:

A. B. [die mensis et anni] pro gradu Baccalaurei in Medicina examinatus, prout Statuta requirunt satisfecit nobis Examinatoribus.

*Ita testamur* { *C. D. P. M. R.*<br>*E. F.*<br>*G. H.* }

Nomina quoque eorum qui Examinatoribus satisfecerunt in Registrum peculiare inserentur penes Registrarium Universitatis, finita quaque Examinatione, adservandum.

Quod si contingat aliquem hujusmodi Testimonio indignum reperiri, licebit illi in aliqua sequente Examinatione Candidatum se iterum profiteri.

Huic Examinationi interesse licebit omnibus Magistris in Artibus, Baccalaureis in Jure Civili, et quibuslibet superiore aliquo Gradu insignitis; necnon Baccalaureis in Artibus, si modo coram Professor Regio spospondérint Medicinæ se operam daturos. Sub eadem conditione licebit Juristis et aliis nondum graduatis, qui examen pro Gradu A. B. vel J. C. B. subierint, et qui quadriennium a die Matriculationis suæ compleverint, his Examinationibus interesse.

Unusquisque Candidatus examen subiturus, Professorem Medicinæ Regium hac de re certiorem facere tenebitur decimo quarto die ad minimum ante Hebdomadam Examinationi habendæ destinatam. Quo quidem tempore, Professori in manus literas certificatorias tradendas curabit, quibus se, apud quoddam melioris notæ Nosocomium, cum morbis curandis interfuisse, tum Lecturis audiendis diligentem operam dedisse, liquido constet. Nec Professori Regio licebit, literis Certificatoriis istis a majori parte Examinatorum non approbatis, aliquem ad examen subeundum admittere.

Cautum insuper esto ne quis se sistat examinandum, nisi qui septem annos (scil. 28 Terminos) a tempore matriculationis suæ compleverit.

§ 4. *De examinatoribus remunerandis.*—Statutum est, quod Candidatus unusquisque ante Examinationem inchoandam Professore Regio in manus deponet sex libras, ab eo, honorarii nomine, duobus reliquis, examinatoribus persolvendas. Ipse Professor feoda antiquitus imperata solus percipiat.

§ 5. *Quot anni, in studio Medicinæ ponendi, ad incipiendum in Medicina requirantur.* Statutum est, quod qui ad Doctoratum in Medicina promoveri cupit, post Gradum Baccalaurei in Medicina susceptum, per tres annos integros studio Medicinæ incumbat, priusquam ad incipiendum in eadem Facultate admittatur.

§. 6. *Exercitium pro Gradu Doctoris in Medicina præstandum.*—Statutum est, quod Baccalaureus in Medicina priusquam ad incipiendnm in eadem Facultate admittatur, dissertationem a se conscriptam de thesi quavis Medica prius a Professore Regio approbata publice intra Scholarum præcinctus coram Professore Regio recitare, eique ejusdem dissertationis exemplar, finita lectione, tradere tenebitur.

Denique ad tollendam omnem dubitationem pronunciamus quod omnes qui in Medicina inceperint, eodem suffragandi jure gaudeant, ac si in Artibus aliquando rexissent.

§. 7. *De incorporandis sive Medicinæ studiosis, sive Graduatis.* Statutum est, quod unusquisque sive Graduatus in Medicina, sive studiosus, ex alia Academia hic incorporandus, priusquam in matriculam hujus Academiæ referatur-testimonia coram Vice-Cancellario exhibeat, quibus liquido pateat eum exercitia præstitisse omnia, quæ in sua Academia nondum Graduati pro Bacca, laureatu in Artibus præstare tenentur. Cautum sit insuper nequis iu Medicina Graduatus incorporetur, nisi examen prius subierit, tempus compleverit, et reliqua præstiterit omnia, quæ per præsens hoc Statutum requiruntur.

SECTIO III.

§. 4. *Formulæ speciales Gratiarum ad singulos pertinentium. Pro Gradu Baccalaurei in Medicina,*—Supplicat, &c. A. B. Magister [vel Baccalaureus] Facultatis Artium, [vel Baccalaureus in Jure Civili, vel Medicinæ studiosus,]

e Coll. (vel Aula) M. quatenus 28 Terminos a die matriculationis suæ compleverit; per triennium Medicinæ operam dederit, examen subierit, et reliqua omnia præstiterit, quæ per Statuta requiruntur, (nisi quatenus secum dispensatum fuerit,) ut hæc sibi sufficiant, quo admittatur ad lectionem cujuslibet libri Aphorismorum Hippocratis.

*Pro Gradu Inceptoris in Medicina.*—Supplicat, &c. A. B. Medicinæ Baccalaureus, e Collegio (vel Aula) N. quatenus post susceptum Gradum Baccalaurei in Medicina, tres annos in studio Medicinæ posuerit; dissertationem scripserit coram Professore recitaverit, et reliqua omnia præstiterit, quæ per Statuta requiruntur, (nisi quatenus secum dispensatum fuerit,) ut hæc sibi sufficiant ad incipiendum in eadem Facultate.

SECTIO IX.

§. 4. *De Qualitate eorum, qui ad Praxin in Medicina licentiandi sunt.* Statutum est, quod Doctor quilibet in Medicina, post inaugurationem seu admissionem suam, practicare licite poterit in omni medicandi genere. Alius vero nemo in Medicina publice practicare Oxoniæ permittatur, nisi Gradum Baccalaurei in Medicina susceperit, et a Cancellario sive ejus Commissario, et Congregatione Magistrorum Regentium, ad practicandum more consueto admissus fuerit. Chirurgiam vero nullus exerceat, intra præcinctum Universitatis, nisi licentia a Cancellario sive Vice-Cancellario impetrata.

Quod si quis secus præsumserit, non solum ab ulteriore promotione repellatur, et privilegiis Universitatis privetur; sed etiam (si monitus non desistat) sicut perturbator pacis puniatur.

§. 5. *Formula petendi Licentiam ad practicandum in Medicinæ.*—Pro qualitate personæ supplicantis, in Gratia exprimantur, quæ ad hujusmodi licentiam necessario requiruntur, sub hac formula: Supplicat, &c. A. B. e Coll. [vel Aula] N., quatenus in hac Universitate Gradum Baccalaurei in Medicina susceterit, et chirographo vel professoris et unius alterius Doctoris in Medicina, vel trium quorumcunque Doctorum in Medicina, in Universitate residentium, approbatus fuerit; et reliqua præstiterit omnia, quæ per Statuta requiruntur, ut hæc sibi sufficiant ad practicandum in eadem Facultate per universam Angliam.]

Qua Gratia concessa et pronunciata, prout aliæ solent, Literæ etiam Testimoniales, de eadem fient, et (eodem modo quo testimoniales licentiæ ad prædicandum in Domo Congregationis ratæ habebantur) sigillo publico Universitatis munientur.

§. *Formula Literarum Testimonialum.*—Cancellarius, Magistri, et Scholares Universitatis Oxon. dilecto nobis in Christo A. B. Baccalaureo in Medicina e Coll. [vel Aula] N. intra Universitatem prædictam, salutem in Domino sempiternam. Cum omnia nostra studia, consilia, et actiones ad Dei gloriam et fratrum salutem referri debeant; cumque Medicina ad hæc, inter reliquas Facultates, plurimum conferat; hinc est, quod nos Cancellarius, Magistri, et Scholares antedicti (pro ea opinione, quam de scientia tua, vitæque ac morum integritate, habemus) liberam tibi, tenore præsentium, concedimus potestatem et facultatem practicandi in Medicina, et ea omnia faciendi, quæ ad eam spectant Facultatem, ubivis per universum Angliæ regnum, in perpetuum duraturum. Nos etiam Cancellarius, Magistri, et Scholares antedicti, testamur præfatum A. B. juramentum de primatu Regiæ Majestatis suscepisse, &c. prout supra, in forma licentiæ ad publice concionandum.

Placuit Academiæ ut hæc Statuta vim et vigorem suum non obtineant ante Terminum Trinit. 1834.

Interim antiqua exercitia rata permaneant.

De Statutis his rogandis consenserunt Collegiorum et Aularum Præfecti, et, re mature perpensa, convenerunt in Terminos, die vicesimo sexto mensis Novembris; relata sunt in Domo Congregationis die vicesimo octavo mensis Novembris, juxta statuti in ea parte exigentiam, triduo ante Convocationem habendam; tandem in Convocatione publicata et confirmata die secundo mensis Decembris, anno Domini 1833.

